# Dietary Diversity Was Positively Associated with Psychological Resilience among Elders: A Population-Based Study

**DOI:** 10.3390/nu11030650

**Published:** 2019-03-18

**Authors:** Zhaoxue Yin, Melanie Sereny Brasher, Virginia B. Kraus, Yuebin Lv, Xiaoming Shi, Yi Zeng

**Affiliations:** 1 Division of Non-Communicable Diseases Control and Community Health, Chinese Center for Disease Control and Prevention, Beijing 102206, China; yinzx@chinacdc.cn; 2 Department of Sociology & Anthropology, Department of Human Development & Family Studies, University of Rhode Island, Kingston, RI 02881, USA; mbrasher@uri.edu; 3 Duke Molecular Physiology Institute and Division of Rheumatology, Department of Medicine, Duke University School of Medicine, Durham, NC 27701, USA; kraus004@duke.edu; 4 Institute of Environmental Health and Related Product Safety, Chinese Center for Disease Control and Prevention, Beijing 100021, China; lvyuebin@nieh.chinacdc.cn; 5 Center for the study of Aging and Human Development and the Geriatric Division of School of Medicine, Duke University, Durham, NC 27701, USA; 6 Center for Healthy Aging and Development Studies, National School of Development, Peking University, Beijing 100871, China

**Keywords:** dietary diversity, psychological resilience, elderly, oldest old

## Abstract

The association between dietary diversity (DD) and psychological resilience among older people is an underdeveloped area of research. This cross-sectional study explored the associations of DD with psychological resilience among 8571 community-based elderly individuals. The intake frequencies of food groups were collected, and dietary diversity was assessed based on the mean DD score. Psychological resilience was assessed using a simplified resilience score (SRS). Data were analyzed using multiple linear regression and logistic regression models. Poor DD was significantly associated with psychological resilience, with a β (95% CI) of −0.94 (−1.07, −0.81) for the SRS (*p* < 0.01) and an odds ratio (95% CI) of 1.83 (1.66, 2.01) for low SRS status. The interaction effects of age with DD were observed for the SRS (*p* < 0.001) and low SRS status (*p* < 0.001). Based on separate analyses by age group, the association of a low SRS with poor DD was more prominent in the younger elderly than the oldest old, with OR (95% CI) 2.32 (1.96, 2.74) and 1.61 (1.43, 1.82), respectively. Compared with younger participants with good DD, the risk of a low SRS was greater for younger participants with poor DD, the oldest old with good DD, and the oldest old with poor DD, with OR (95% CI) 2.39 (2.02, 2.81), 1.28 (1.09, 1.51), and 2.03 (1.72, 2.39), respectively. The greatest contribution to DD was from a high consumption of vegetables, fruits, and nuts. Our study suggested that poor DD was associated with a low psychological resilience among the Chinese elderly, especially the younger elderly. These findings suggest that augmentation of DD might promote psychological resilience.

## 1. Introduction

Psychological resilience is defined as the ability to cope, adapt, and respond positively to stress or adversity [1,2]. Adversity is deemed a primary antecedent of resilience; positive adaptation is considered a consequence of resilience [3]. Several studies have shown an association of psychological resilience with healthy aging. For example, studies have suggested that high psychological resilience predicts greater happiness, lower depression [4,5], and a greater satisfaction with life [6]. Resilience is also positively correlated with self-rated successful aging [7]. Higher resilience is associated with positive physical outcomes, such as activities of daily living (ADL) [8], physical health [9], as well as exceptional longevity and survival [10,11]. Given that the world’s older population is increasing dramatically and the aging of the population has become a big challenge for public health, studies of factors associated with psychological resilience in elderly individuals are of great importance.

Resilience is considered as a dynamic process, which involves the interactions of factors related to individual, community, and societal levels [12]. Factors associated with positive and negative adaptation in the face of adversity can influence psychological resilience [13]. On an individual level, psychological factors, such as self-efficacy and internal locus of control [14], biological factors, which include physical health and genetic factors [15,16], health behaviors, for example exercise [17], as well as some social factors are associated with psychological resilience.

Adaptation to adversity may be influenced by dietary factors—one kind of health behavior. Some studies have shown positive associations of specific healthy eating behaviors with specific personality traits, reflecting psychological resilience [18]. Associations have also been demonstrated between specific plant chemical substances and psychological resilience [19]. Based on limited studies, dietary pattern, such as the Mediterranean-type dietary pattern and the vegetable-based dietary pattern, and diet quality are associated positively with psychological resilience among the general population (mean age of 52.7 years) or young adults (mean age of 21.0 years) in developed countries [20,21]. However, studies reporting direct associations of dietary diversity (DD) with psychological resilience are rare, especially among older people in undeveloped countries.

DD, defined as the number of different food groups consumed during a given reference period [22], is a key element of high-quality diets and is recommended in the dietary guidelines of many countries. Among elders, DD is associated with nutrient adequacy and health status, including cognitive function [23,24]. Studies have also shown that a diet rich in nutrients may be protective of mental health [25], so we hypothesize that DD may be associated with psychological resilience among older people. DD can be considered as one of the intervention strategies of promoting resilience if the association of DD with psychological resilience is established. However, the age-related associations of DD and psychological resilience have not previously been investigated, although an older age was associated with a higher resilience [13,26].

Due to its potential public health implications for healthy aging, the goal of our study was to explore the association of DD with psychological resilience across a range of adult age groups. In this study, we pursued these goals among the Chinese elderly using the community-based Chinese Longitudinal Healthy Longevity Survey (CLHLS).

## 2. Materials and Methods

### 2.1. Study Subjects

Participants for this study were aged 65 years and above, ascertained during the sixth wave of the Chinese Longitudinal Healthy Longevity Survey (CLHLS) conducted in 2011–2012. The CLHLS was the first national longitudinal study on the determinants of healthy aging in China, which collected data from a very large community-based population sample of the oldest old (age of 80 years and over) and younger elderly (age of 65–79 years). The details of the study design and its good data quality have been described in a previous publication [27]. Of the 9765 people who participated in the 2011–2012 wave survey, 1607 were excluded due to missing data on some key variables, such as resilience and dietary intake frequency. Thus, 8158 elderly people, including 3023 younger elderly and 5135 oldest old, were analyzed in this study.

The CLHLS was approved by the ethics committee of Peking University, and written informed consents were obtained from all participants (or their proxies).

### 2.2. Psychological Resilience Assessment

As described previously [11], the simplified resilience score (SRS) was used to assess psychological resilience in this study. Briefly, the SRS was constructed according to the general theory framework of the Connor–Davidson Resilience Scale (CD-RISC) [28]. The SRS includes seven questions related to psychological resilience, which reflect important factors of resilience with counterparts in the CD-RISC, including personal tenacity, optimism, coping with negative mood, secure relationship, and self-control. The total SRS ranges from 0 to 22, and higher scores indicate a better psychological resilience. Consistent with our previous study [11], a high resilience was defined as a SRS score ≥16.

### 2.3. Assessment of Dietary Diversity

In the CLHLS, the consumption frequencies of various food groups, including vegetables, fruits, legumes and their products, nuts, meat, eggs, fish, dairy and its products, tea, as well as cereals and oil, were asked for. As reported in our previous study [24], DD was constructed using the first 9 food groups, based on the principle that the selection of food groups for assessing DD can be driven by the specific purpose [22]. Briefly, if the response for one food group was once per week at least, one point was given, otherwise no point was given. Then the points of all nine food groups mentioned above were totaled, so the total dietary diversity score (DDS) ranged from 0 to 9. A higher DD score reflected better dietary diversity. As suggested by FAO [29] and used in our previous study [24], DD was defined as ‘poor’ if the DDS was lower than the mean value; DD was defined as ‘good’ for scores at or above the mean.

### 2.4. Covariates

Information on covariates was collected, including sociodemographics (age, sex, education level, and marital status), lifestyles (smoking, alcohol drinking, and physical activities), leisure activities, social activities, waist circumference, blood pressure, hearing decline, ADL disability, diabetes, and stroke history. These covariates were defined as in our previous CLHLS study [24]. Briefly, physical activities were defined as ‘yes’ if the response for physical exercise or outdoor activities was ‘yes’. Leisure activities, which included growing flowers, raising pets, reading books, playing cards, watching TV, and listening to the radio, were defined as ‘yes’ if the frequency of any of these items was once per week or more. Social activities were defined in the same way as leisure activities [30]. Systolic blood pressure (SBP) and diastolic blood pressure (DBP) were measured twice on the right arm, and the mean values were used. Abdominal obesity was defined based on waist circumference using the following criteria: ≥90 cm for males and ≥85 cm for females. Hearing decline was assessed using the question, ‘Has your hearing declined in recent years?’ ADL was assessed by the Katz Activities of Daily Living Scale [31].

### 2.5. Statistical Analysis

Participants’ characteristics by psychological resilience status (low and high) were compared by t-test for continuous variables and by chi-square test for categorical variables. To explore the associations of poor DD with psychological resilience, general linear regression analysis was used to estimate the β coefficient and the 95% confidence interval (CI) of poor DD for the SRS after confirming the normality of residuals in the general linear models. Logistic regressions were used to analyze the odds ratio (OR) and 95% CIs of poor DD for low SRS status, for which the SRS and low SRS status were used as dependent variables, respectively. Three models were adjusted as follows: Model 1 adjusting for demographic variables (i.e., age, sex, education, and marital status), Model 2 further adjusting for smoking, alcohol drinking, physical activities, leisure activities, and social activities, and Model 3 further adjusting for hearing decline, ADL disability, stroke, and diabetes.

To explore whether the associations of DD with psychological resilience varied with age, the interaction effects of DD with age groups (younger elderly and oldest old) on the SRS and low SRS status were assessed. When potential significant statistical interactions were detected, the associations of DD with psychological resilience were investigated among the younger elderly and oldest old separately. Using the group of younger elderly with good DD as the reference group, we also classified the participants into four groups—younger elderly with good DD, younger elderly with poor DD, oldest old with good DD, and oldest old with poor DD—to assess the joint effect of age group and DD on psychological resilience.

According to the method used in previous studies [20,32], we also assessed the relative contributions of the nine food groups included in DD in relation to psychological resilience using a ‘leave one out’ method. Briefly, to assess the relative effect of various food groups, all nine food groups, coded dichotomously, were simultaneously included in a multivariate linear regression model, and the variance inflation factors obtained in the model suggested there were no significant collinearity problems. We then removed one food group at a time from the total SRS to obtain β coefficients associated with the new DD score deprived of this food group. We thus reduced the 10 scores (0–9 points) to 9 scores, producing an estimation of the change in magnitude of associations of psychological resilience with each one unit change in the score, for instance, minus vegetables, minus fruits, minus legumes and their products, minus nuts, minus meat, minus eggs, minus fish, minus dairy and its products, and minus tea.

All statistical analyses were performed with SAS, version 9.2 (SAS Institute Inc., Cary, NC, USA). A *p* < 0.05 was considered statistically significant; all *p* values were two-sided.

## 3. Results

The characteristics of the participants by resilience status are listed in Table 1. Compared with those with low resilience scores, those with high resilience scores were younger, more likely to be educated, married, and usually reported more physical activities, leisure activities, social activities, and a technology-related occupation history, in addition to a lower prevalence of hearing decline and ADL disability. The mean DD score was higher for those with a higher SRS compared to subjects with a lower SRS (4.90 versus 3.95).

Compared with good DD, poor DD was significantly associated with low resilience, with a β (95% CI) of −0.94 (−1.07, −0.81) for the SRS (*p* < 0.01), and an odds ratio (95% CI) of 1.83 (1.66, 2.01) for lower SRS status in Model 3 (Table 2).

We also observed a statistically significant interaction of age with DD on lower SRS status (*p* interaction < 0.001) and the SRS (*p* interaction = 0.001). In separate analyses, poor DD was significantly associated with the SRS in both the younger elderly and the oldest old (*p* < 0.01), with a β (95% CI) of −1.14 (−1.35, −0.94) and −0.80 (−0.97, −0.64), respectively. Poor DD was also significantly associated with lower SRS status, with odds ratios (95% CI) of 2.32 (1.96, 2.74) in the younger elderly and 1.61 (1.43, 1.82) in the oldest old (Table 3).

**Figure 1:**
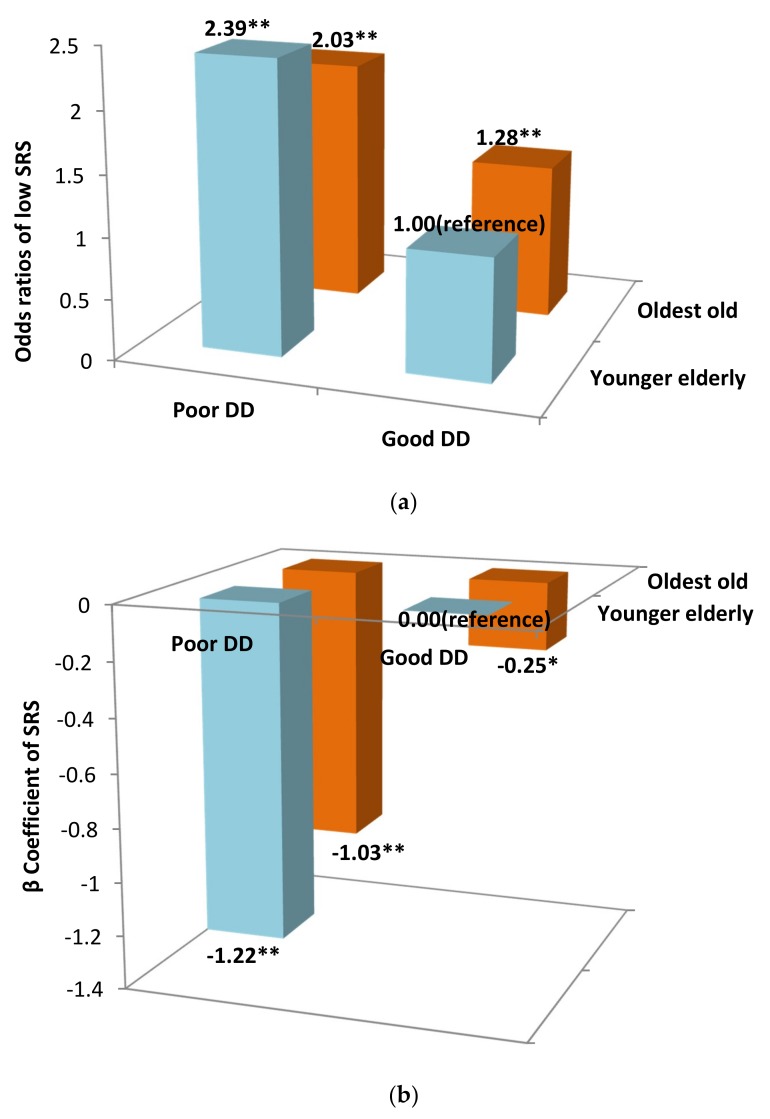
(**a**) The joint effect of age group and DD on a low SRS, with the younger elderly with good DD as the reference group. Abbreviations: CI—confidence interval; DD—dietary diversity; SRS—simplified resilience score; OR—odds ratio. The results were adjusted for sex, education level, marital status, smoking, alcohol drinking, physical activities, leisure activities, social activities, hearing decline, ADL disability, stroke history, and diabetes. ** *p* < 0.01. (**b**) The joint effect of age group and DD on the SRS, with the group of younger elderly with good DD as the reference group. Abbreviations: CI—confidence interval; DD—dietary diversity; SRS—simplified resilience score; OR—odds ratio. The results were adjusted for sex, education level, marital status, smoking, alcohol drinking, physical activities, leisure activities, social activities, hearing decline, ADL disability, stroke history, and diabetes. * *p* < 0.05, ** *p* < 0.01.

Compared with younger participants with good DD, the risks of a low SRS for the younger elderly with poor DD, the oldest old with good DD, and the oldest old with poor DD all increased significantly but differently, with an OR (95% CI) of 2.39 (2.02, 2.81), 1.28 (1.09, 1.51), and 2.03 (1.72, 2.39), respectively. The SRS also decreased significantly and differently in these groups, with a β (95% CI) of the SRS for the younger elderly with poor DD, the oldest old with good DD, and the oldest old with poor DD of −1.22 (−1.42, −1.01), −0.25 (−0.45, 0.05), and −1.04 (−1.24, −0.83), respectively (Figure 1a,b).

When the nine food groups were included simultaneously in the full model, only fish and tea were not associated with psychological resilience (*p* > 0.05); the remaining seven food groups, especially vegetables and fruits, were all significantly associated with psychological resilience (*p* < 0.01) (Table 4). As shown in Table 5, the greatest reduction in psychological resilience was observed upon removing vegetables, fruits, and nuts from the DD score, and the reduction in the β coefficients was 9.31%, 8.97% and 6.9%, respectively (Table 5).

## 4. Discussion

To the best of our knowledge, this is the first study focusing on associations between dietary diversity and psychological resilience among older adults, our finding of the positive association of poor DD with a lower SRS among elders has added novel knowledge on improving psychological resilience.

Although studies specifically addressing the associations of DD with psychological resilience are very scarce, our findings are supported by other limited previous studies investigating associations of a predefined dietary pattern or empirically derived dietary patterns with resilience. For instance, greater adherence to Greek Mediterranean diets and an ‘olive oil and vegetable’ dietary pattern was positively associated with psychological resilience, with a β (95%CI) of 0.43 (0.19, 0.66) and 1.18 (0.93, 1.44), respectively [20]. In addition, a 2010 healthy eating index (HEI-2010) was correlated with psychological resilience; a higher healthy eating index (HEI) predicted an increased likelihood of having a higher resilience [21]. However, the assessment of dietary pattern is relative complicated. Compared with assessing dietary pattern, the calculation of DD is easier and more applicable in population surveillance or in an intervention program, and therefore DD is much more readily instituted as part of a strategy to improve psychological resilience in elders.

Although the mechanism underlying it was not clear, there are several possible explanations for the association of DD with psychological resilience. The DD score is a useful proxy of nutrient adequacy and better nutritional status [23,33], so good DD may predict a higher resilience. In fact, good nutritional status can potentially improve resilience, as shown for patients with chronic kidney disease [34]. However, poor DD is usually characterized by poor nutrient intake. Since some nutrients need enough other nutrients from various food groups to perform their role, the effects of DD on resilience can likely only be observed in the presence of high DD [24]. Second, low DD is correlated with a high level of oxidative stress [35]. Oxidative damage to mitochondria and lipids in neuronal circuits is associated with affective behaviors [36]; hence low DD may entail a high risk of low resilience.

Consistent with other previous studies [20], we observed that vegetables, fruits, and nuts contributed the most to the SRS. Dietary polyphenols, a group of phenolic compounds abundant in fruits, vegetables, and other plant sources, may be beneficial for psychological resilience [19]. Women with a resilient personality were more likely to report a higher intake of vegetables and fruits [18]. In another study, resilient people were more likely to consume more than five servings of fruit and vegetables a day [37]. Nut consumption can favorably modulate the key factors in depressive symptoms, such as inflammation and oxidation [38]. It has been concluded that nut consumption is associated with a higher resilience [20], due to their favorable fatty acid profile characterized by a high content of unsaturated fat (nearly half of the total fat) and monounsaturated fatty acids (MUFAs).

Another new interesting finding in our study was the interaction effect of age and poor DD on resilience, i.e., the association of DD with resilience was influenced by age. We found a larger coefficient of poor DD among the younger elderly. A previous study found that the oldest old, aged 85 and over, appeared to have the same or greater capacity for psychological resilience than those who were younger [39], and this study suggested that the greater negative effect of poor DD on psychological resilience in the younger elderly would be one of the possible reasons. This result is supported by the inoculation theory of resilience, that is, life experience and increased opportunities for exposure to stressful events inoculates the individual against adversities [40]. This also supports Carstensen’s socio-emotional selectivity theory, that even with potentially reduced social networks, older adults focus on more meaningful emotional goals [41]. Compared with the younger elderly, the oldest old experienced more adversities and so strengthened the skills to cope with them, thereby their resilience was less influenced when DD was poor.

Some strengths of the study also include the large sample size and number of covariates including the social factor, i.e., social activities. However, our findings should be interpreted with caution. First, the cross-sectional design of this analysis could not infer a causal relationship. Reverse causality should be considered—low resilience could result in changes of food choices; moreover, individuals with a high resilience may be more likely to prepare a diet with good DD. Second, the assessment of DD was based on the consumption frequency of specific food groups, not on the standard food frequency questionnaire—this may limit the generalizability of our results. However, food group diversity is a user-friendly methodology with the advantage of simplicity and, as shown previously [22], appropriate for capturing the major factors related to DD.

This large-scale population-based study suggests that dietary diversity is positively and significantly associated with psychological resilience among the elderly, with larger coefficients among the younger elderly subsample. These findings have important public health implications. Interventions aiming to improve DD may be beneficial for psychological resilience, and physicians may encourage older people to improve dietary diversity, given that DD is very simple and easy to be applied. Future work is necessary to further assess these findings in longitudinal studies and experimental trials.

## Figures and Tables

**Table 1:** Characteristics of study participants by psychological resilience status.

Characteristics	Total Sample	SRS Status		
		Low	High	*p* Value
No. of subjects	8158	3555	4603	
Age (years), mean (SD)	84.90 (10.84)	86.76(10.67)	83.45 (10.75)	<0.001
Female	4318 (52.93)	2142 (60.25)	2176 (47.27)	<0.001
Education years				
0	4499 (55.15)	2342 (65.88)	2157 (46.86)	<0.001
1–6	2652 (32.51)	944 (26.55)	1708 (37.11)	
>6	1007 (12.34)	269 (7.57)	738 (16.03)	
Married	3394 (41.60)	1164 (32.74)	2230 (48.45)	<0.001
Technical occupation history	1591 (19.50)	457 (12.86)	1134 (24.64)	<0.001
Smoking	1553 (19.04)	556 (15.64)	997 (21.68)	<0.001
Alcohol drinking	1431 (17.54)	479 (13.47)	952(20.68)	<0.001
Physical activities	4536 (55.60)	1597 (44.92)	2939 (63.85)	<0.001
Leisure activities	6057 (74.25)	2235 (62.87)	3822 (83.03)	<0.001
Social activities	4614 (56.56)	1763(49.59)	2851 (61.94)	<0.001
Stroke	1098 (13.46)	486 (13.67)	612 (13.30)	0.62
Diabetes	1054 (12.92)	429 (12.07)	625 (13.58)	0.04
ADL disability	1710 (21.36)	992 (28.44)	718 (15.89)	<0.001
Hearing decline	3467 (42.6)2	1754 (49.46)	1713 (37.34)	<0.001
DDS, mean (SD)	4.48 (1.99)	3.95(1.95)	4.90 (1.93)	<0.001
Poor DD	4056 (49.72)	2163(60.84)	1893(41.13)	<0.001
SRS	1593 (3.06)	13.11 (1.87)	18.11 (1.75)	<0.001

Abbreviations: ADL—activities of daily living; DD—dietary diversity; DDS—dietary diversity score; SD—standard deviation; SRS—simplified resilience score. Data were shown as n (%) for categorical variables and shown as mean (SD) for continuous variables.

**Table 2:** Association of DD with SRS and low SRS (*n* = 8158).

DD	Model 1	Model 2	Model 3
β coefficient (95% CI) of SRS			
Good	0.00 (reference)	0.00 (reference)	0.00 (reference)
Poor	−1.08 (−1.21, −0.95) **	−0.92 (−1.06, −0.79) **	−0.94 (−1.07, −0.81) **
Odds ratio (95% CI) of low SRS status			
Good	1.00 (reference)	1.00 (reference)	1.00 (reference)
Poor	1.93 (1.76, 2.13) **	1.80 (1.63, 1.98) **	1.83 (1.66, 2.01) **

Abbreviations: DD—dietary diversity; CI—confidence interval; SRS—simplified resilience score. Model 1: adjusting for age, sex, education, and marital status; Model 2: additionally adjusting for smoking, alcohol drinking, physical activities, leisure activities, and social activities; Model 3: additionally adjusting for health status, including hearing decline, activities of daily living (ADL) disability, stroke history, and diabetes. ** *p* < 0.01.

**Table 3:** Association of DD with SRS and lower SRS status among the younger elderly and the oldest old.

DD	Younger Elderly (*n* = 3023)		Oldest Old (*n* = 5135)	
	Model 1	Full model	Model 1	Full model
β coefficient (95% CI) of SRS				
Good	0.00 (reference)	0.00 (reference)	0.00 (reference)	0.00 (reference)
Poor	−1.33 (−1.53, −1.12) **	−1.14 (−1.35, −0.94) **	−0.94 (−1.10, −0.77) **	−0.80 (−0.97, −0.64) **
OR (95% CI) of Low SRS status				
Good	1.00 (reference)	1.00 (reference)	1.00 (reference)	1.00 (reference)
Poor	2.48 (2.11, 2.92) **	2.32 (1.96, 2.74) **	1.71 (1.52, 1.92) **	1.61 (1.43, 1.82) **

Abbreviations: DD—dietary diversity; OR—odds ratio; CI—confidence interval; SRS—simplified resilience score. Model 1: adjusting for age, sex, education, marital status, and occupation history; Full model: additionally adjusting for smoking, alcohol drinking, physical activities, leisure activities, social activities, hearing decline, activities of daily living disability, stroke history, and diabetes (Model 3). ** *p* < 0.01.

**Table 4:** Mutually adjusted associations of food groups used in DDS with psychological resilience in multivariate linear regression.

Food Group	β Coefficients (95% CI)	*p* Value
Meat	0.418 (0.267, 0.570)	<0.001
Bean	0.263 (0.129, 0.397)	0.001
Vegetables	0.489 (0.287, 0.692)	<0.001
Fruits	0.558 (0.420, 0.695)	<0.001
Egg	0.304 (0.157, 0.451)	<0.001
Fish	0.039 (−0.097, 0.175)	0.574
Milk	0.298 (0.154, 0.442)	<0.001
Tea	0.058 (−0.081, 0.198)	0.412
Nut	0.302 (0.109, 0.495)	0.002

Abbreviations: CI—confidence interval; DDS—dietary diversity score. The results were adjusted for sex, education level, marital status, smoking, alcohol drinking, physical activities, leisure activities, social activities, hearing decline, ADL disability, stroke history, and diabetes.

**Table 5:** Change in the magnitude of associations of ‘leave one out’ DDS with SRS when each of the food groups was removed.

DDS	β Coefficients (95% CI)	Change
Total DDS	0.290 (0.257, 0.323)	
Minus meat	0.275 (0.242, 0.308)	−5.17%
Minus beans	0.288 (0.255, 0.322)	−0.70%
Minus vegetables	0.263 (0.231, 0.294)	−9.31%
Minus fruits	0.264 (0.230, 0.297)	−8.97%
Minus egg	0.285 (0.252, 0.319)	−1.72%
Minus fish	0.302 (0.269, 0.336)	4.13%
Minus milk	0.277 (0.244, 0.310)	−4.48%
Minus tea	0.286 (0.255, 0.319)	−1.38%
Minus nuts	0.270 (0.239, 0.302)	−6.90%

Abbreviations: CI—confidence interval; DDS—dietary diversity score. The results were adjusted for sex, education level, marital status, smoking, alcohol drinking, physical activities, leisure activities, social activities, hearing decline, ADL disability, stroke history, and diabetes.
